# Molecular Fingerprinting and Phytochemical Investigation of *Syzygium cumini* L. from Different Agro-Ecological Zones of India

**DOI:** 10.3390/plants12040931

**Published:** 2023-02-17

**Authors:** Suphiya Khan, Swati Agarwal, Krati Singh, Anil Chuturgoon, Ashutosh Pareek

**Affiliations:** 1 Department of Bioscience and Biotechnology, Banasthali Vidyapith, Banasthali 304022, India; 2 Drumlins Water Technologies Pvt. Ltd., Jaipur Rajasthan 302005, India; 3 Discipline of Medical Biochemistry, School of Laboratory Medicine and Medical Sciences, University of KwaZulu-Natal, Durban 4041, South Africa; 4 Department of Pharmacy, Banasthali Vidyapith, Banasthali 304022, India

**Keywords:** *Syzygium cumini*, agro-ecological zones, DNA fingerprinting, TLC, HPLC, ISSR markers, polyphenols, pharmaceutical

## Abstract

*Syzygium cumini* L. (ver Jamun; BlackBerry) is a native, evergreen multipurpose tree species of India. Besides being a fruit tree and for agroforestry in different regions, it is medicinally important too. This study aimed to determine genetic diversity using molecular and phytochemical markers in sixteen genotypes of Indian *S. cumini* from different agro-ecological zones. The present study used a combination of ISSR markers and the HPLC technique to explore these genotypes. The results showed a wide genetic diversity range based on the similarity coefficient values observed in *S. cumini* sixteen accessions from different sites. Four primary phenolic acids were discovered in all the accessions; caffeic acid (CA) was found in high concentrations. The intraspecific association between molecular and phytochemical characteristics was the primary goal of this investigation. By employing gene-specific markers for the route of secondary metabolites (polyphenols) production, it further investigated the progressive research of diversity analysis of polyphenol content in *S. cumini* accessions, which may also expand its nutraceutical and pharmaceutical utilization.

## 1. Introduction

Biodiversity loss is now one of the world’s most pressing crises, and parts of the world endowed with rich genetic resources are facing grim scenarios with the alarming rate of loss of biodiversity. IUCN recognizes inadequate coverage of evaluations across all plant categories to report an accurate percentage of vulnerable species for plants. In total, 54% of plant species are currently recognized on the IUCN Red List as threatened [1]. Further, more than half of the vascular plant species in the world are currently found in 35 hotspots [2]. Ecosystem diversity, species diversity, and genetic diversity are the three categories used to categorize biological variety, and efforts are being made at all levels to conserve biodiversity. In addition to being essential for ecological and evolutionary research, the sum and circulation of genetic diversity within and among populations also form the basis for functional genomic investigations [3].

*Syzygium cumini* L. (ver Jamun; BlackBerry), a native, evergreen tree species of India, thrives simply in a tropical climate and is found not only in numerous parts of the Indian sub-continent but also several other countries of Asia and Eastern Africa [4]. In India, the tree is widely grown in the Indo-Gangetic plains and the Cauvery delta of Tamil Nadu [5]. Besides being important as a fruit tree and for agroforestry in different regions, *S. cumini* is medicinally important too, and over many decades, traditional practitioners have employed various methods to manage diabetes [6]. Despite having a high economic worth, nothing is known about this species’ reproductive habits, population genetic makeup, or intraspecific variation across its range.

Various methods can be used to measure a species’ genetic diversity. During the past several decades, comparative anatomy, morphology, embryology, and physiology, as well as other traditional methods of assessing genetic diversity, have been increasingly supported by molecular approaches [7]. Quick surveys of genetic diversity in and among inhabitants have been made possible by the recent rapid development of biochemical and molecular markers. DNA polymorphism is measured using several molecular markers, categorized as polymerase chain reaction markers and hybridization-based markers. The inter-simple sequence repeat (ISSR) method uses areas oriented in the opposing strands to amplify the DNA segment at the amplifiable distance between two identical microsatellites. Such amplification produces multilocus and highly polymorphic patterns without requiring awareness of the genome sequence [8].

The exact genotype is guaranteed by DNA fingerprinting, but the active ingredient or chemical components are not disclosed. Consequently, as a method for authenticating germplasm, phytochemical variations are combined with genetic analyses [9] and biodiversity evaluation [10]. Molecular phylogenetic and phytochemistry studies provide insights into the development of phytochemical differentiation and the function of secondary metabolites [11]. Among the phytochemicals, phenolics are the largest and most extensively circulated in the plant kingdom. Hydroxycinnamic and hydroxybenzoic acids, which are both commonly used, are within the broad group of phenolic acids. For the qualitative analysis of a limited number of substances, thin-layer chromatography (TLC) and high-performance liquid chromatography (HPLC) are often employed. It is known that geographical factors influence the biological activity profiles of plants of medicinal use and, consequently, their active mechanisms. Such phytochemical variations can be recorded using HPLC [12].

This study collected genetic information from different accessions of *S. cumini* leaves germplasm using ISSR is corroborated. Such research would demonstrate the use of DNA fingerprinting in recognizing the secondary metabolism-related genes in economically significant plants for their breeding programs and, ultimately, for intellectual property rights. This work is the continuation of our previous study in which these germplasms were studied using RAPD markers [13].

## 2. Materials and Methods

### 2.1. Materials

#### 2.1.1. Chemicals and Reagents

Various chemicals and standard DNA molecular weight markers used in fingerprinting studies were of molecular biology grade and obtained from Bangalore Genei Pvt. Ltd. (India). The ISSR primers were custom manufactured from Bangalore Genei Pvt. Ltd. (Karnataka, India). Standards for TLC and HPLC (caffeic acid, ferulic acid, o-coumaric acids, sinapic acid, and p-coumaric acids) were purchased from Fluka Chemica (Taufkirchen, Germany). The solvents used are of HPLC grade and purchased from Rankem (Haryana, India).

#### 2.1.2. Plant Materials

**Figure 1:**
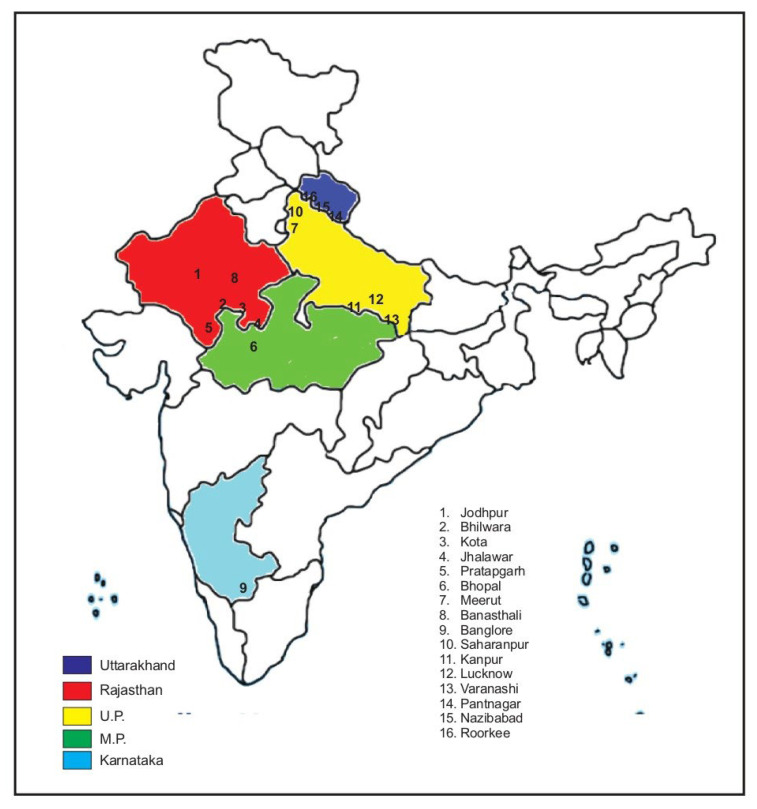
India map showing *S. cumini* accessions gathered from agro-ecological zones.

Fresh and fully expanded three-five leaves of *S. cumini* from one individual wild tree were collected from four primary agro-ecological zones—moist sub-humid, dry sub-humid semi-arid, and moist sub-humid, arid—and nine sub-zones—leaf samples stood gathered from sixteen specific geographic sites, indicating five states of India: Uttarakhand, Madhya Pradesh, Uttar Pradesh, Karnataka, and Rajasthan (Table 1; Figure 1) and were then shed dried up at the site, relocated in a brown paper envelope and sealed.

### 2.2. Method

#### 2.2.1. Genomic DNA Isolation from *S. cumini*

Genomic DNA was extracted using the CTAB procedure with certain modifications [13,14].

#### 2.2.2. Molecular Analysis by Using ISSR Marker

The amount of DNA isolated was measured using agarose gel electrophoresis. Twenty-six ISSR markers (Table 2) were employed in the molecular marker analysis to characterize 16 distinct accessions of *S. cumini* plant samples. ISSR analysis was performed using genomic DNA at a concentration of 25 ng. DNA amplifications were performed using a thermocycler (PQ lab-Primus 96) for the ISSR analysis. One negative control (master mix with water in place of DNA template) was added to check for contamination in the experiment. All the molecular analyses were conducted in three replicates.

A submerged gel electrophoresis midi unit from Bangalore Genei Pvt. Ltd. was used for fractionating the PCR products on an agarose gel. A low-range DNA ruler was included on one side of the gel as a molecular standard. The gel was visualized on a UV transilluminator (Bio-Rad, USA), photographed, and analyzed using the Kodak gel documentation system (Model EDAS 290) using Kodak ID Image analysis software.

#### 2.2.3. Phytochemical Study of *S. cumini*

This study generated a chem profile of different *S. cumini* germplasm with chromatographic techniques TLC and HPLC.

1. Extraction and estimation of polyphenols

The Folin-Ciocalteu technique was expended to ascertain the total polyphenol contented (TPC) [15]. Samples of leaves were made at a concentration of 1 g in 10 mL of buffer. The incubation of the mixture was done for 90 min at 25 °C and analyzed by UV-Vis spectrophotometer at 725 nm.

2. Separation of polyphenols using TLC

For the TLC analysis, standards (caffeic acid, ferulic acid, o-coumaric acids, sinapic acid, and p-coumaric acids) and polyphenol samples were exposed to qualitative TLC analysis on cellulose plates (20 × 20 cm, Merck, Darmstadt, Germany). Extracted polyphenol samples (20 µL) were spotted on the plate and developed under the following mobile phase conditions: (1) benzene: acetic acid: water (37:45:18) (2) ethyl acetate: acetic acid: water (60:20:10) (3) ethyl acetate: methanol: water (10:1.65:1.35) (4) ethyl acetate: methanol: formic acid: water (100:13.5:2.5:10) (5) ethyl acetate: toluene: acetic acid (50:40:20) (6) chloroform: acetic acid (90:10) (7) ethyl acetate: formic acid: acetic acid: water (100:11:11:27) (8) ethyl acetate: acetic acid (80:20) (9) ethyl acetate: methanol (80:20). The established plates remained to soak up at room temperature, protected from light and were assessed under UV light at 254 nm. Further, the developed spots were visualized with general sprays for finding phenols, 2% aqueous FeCl_3,_ and 1% methanolic KOH.

3. Separation of polyphenols using HPLC

Standards (caffeic acid, ferulic acid, o-coumaric acids, sinapic acid, and p-coumaric acids) stock solutions of 1 mg/mL concentration were arranged. The samples were examined using an HPLC system (Shimadzu, LC10A, Kyoto, Japan) with solvents A (2% aqueous glacial acetic acid) and B (30% acetonitrile and 2% aqueous glacial acetic acid), along with an analog SCL 10 AVP pump, an injection (20 µL loop), and an SPD 10 AVP detector. Data acquisition was made using Class VP Software. Chromatography was performed on a C8 column (250 × 4.6 mm; particle size 5 µm; Phenomenex, Torrance, CA, USA). A maximum pressure of 400 kgf/cm^2^ and a minimum of 0 Kgf/cm^2^ were sustained, with a solvent ratio of 30:70 using A and B solvents, respectively, at a wavelength of 320 nm and 1 mL of flow rate with a binary mode of gradient system. Peak areas and retention times were computed using a Pentium computer and a Shimadzu Class-VP integrator.

4. Statistical Analysis

The ISSR data cast off to accumulate a separate binary matrix for cluster examination using the NTSYS-pc 2.1 [16]. The SIMQUAL (Similarity for Qualitative Data) procedure assessed genetic similarity between collections according to Jaccard’s similarity coefficient [17]. The dendrogram was then created using the similarity coefficients and the UPGMA (Unweighted Pair Group Method with Arithmetical Averages) function of the NTSYS-pc 2.1 package’s SAHN (Sequential Agglomerative Hierarchical and Nested Clustering) routine. The dendrogram was graphically signified as a phenetic tree through the TREE program NTSYS-pc 2.1 package. Finally, the binary data based on Nie’s coefficient matrix was used to calculate principal component analysis (PCA) with the same program using the EIGEN and PROJ modules to highlight the resolving power of ordination. The percentage of polymorphic bands (PPB) and resolving power (Rp) were calculated [18]. Rp and polymorphism information content (PIC) or gene diversity value were calculated using the method [19]. According to the indices, the information content of each marker system was established [20]. Conclusively, the gathered data from the molecular and phytochemical analysis of *S. cumini* samples the correlation analysis was performed using XLSTAT-2015 software [21].

## 3. Results and Discussion

### 3.1. Molecular Analysis

#### 3.1.1. DNA Extraction Analysis

Though the plant species are the same, the molecular weight of genomic DNA ranged from 19.5 kb to 20.4 kb (Table 3). Kulkarni et al. [22] reported that isolation of genomic DNA from *S. cumini* was challenging because of high levels of polyphenols, tannins, and polysaccharides, which on cell disruption, form a sticky gelation in which nucleic acids become embedded. However, in the present case, overall, a good yield of genomic DNA was obtained from most of the samples of *S. cumini* collected from different locations. Genetic DNA, yield, and purity variations could be explained as each plant is grown in different climatic conditions, which might result in varying types and amounts of secondary metabolites, reflecting the DNA isolation and yield.

#### 3.1.2. Genetic Diversity Analysis

A set of 26 ISSR primers was utilized for the initial screening of 16 *S. cumini* populations, of which 12 primers gave amplification. However, only 8 primers generated a clear, reproducible pattern (Supplementary Figures S1 and S2). The 8 primers generated 98 unambiguous and reproducible bands, of which 95 (96.25) were polymorphic (Table 4). A high percentage of polymorphic fragments (96.93%) were found in the PCR amplification using ISSR markers. It was predicted that the ISSR approach boosts possibly polymorphic microsatellite regions [23]. The number of bands speckled from 7 (22BV17 T8) to 17 (17BV18 T3), with a mean of 12.25 bands per primer. The size of the amplification product varied from 200–2500 bp. The resolving power of ISSR primers varied from 6.25 (20BV17A6) to 14 (15BV17C1), with an average resolving power of 10.08 (Table 4). The resolving power of primer 15BV17C1 was found to be 14; hence, it could be considered the most informative in terms of resolving power and the capacity of primers to distinguish between all accessions. Thus, it showed that the special price of resolving power designated the capability of primers to resolve the diverse, thoroughly linked accessions of *S. cumini*. Our consequences suggested that dinucleotide and trinucleotide ISSR occur at high frequencies.

#### 3.1.3. ISSR Marker-Based Genetic Similarity and Cluster Analysis

Correspondence indices differentiated all the *S. cumini* accessions and directed a fair variety of unpredictability (0.45 to 0.90) in the resemblance coefficient values, signifying a broad genetic base of 16 genotypes investigated in the present study. Accessions from Saharanpur (CD-5.4) and Meerut (D-4.3) that belonged to dry sub-humid and semi-arid zone, respectively, were found most similar to each other (similarity coefficient 0.89). Similarly, samples collected from neighboring cities of Meerut (D-4.3) and Kanpur (CD-5.4), which too belonged to dry sub-humid and semi-arid zone, respectively, had a high similarity coefficient (0.87). The last value of similarity coefficient (0.45) was detected between the genotypes from Nazibabad (CD-6.1) and Kota (D-4.2), which fitted to moist sub-humid and semi-arid zone, respectively. Similarly, Kota (D-4.2) and Bangalore (D-4.4) belonged to the same zone, i.e., semi-arid had a low similarity coefficient value (0.47) (Table 4). Among sixteen samples of *S. cumini,* genetic similarity values were distributed in an acceptable range and displayed maxima at 0.5 similarity coefficient values. The current study proposes that ISSR is suitable for genetic diversity in tree species.

**Figure 2:**
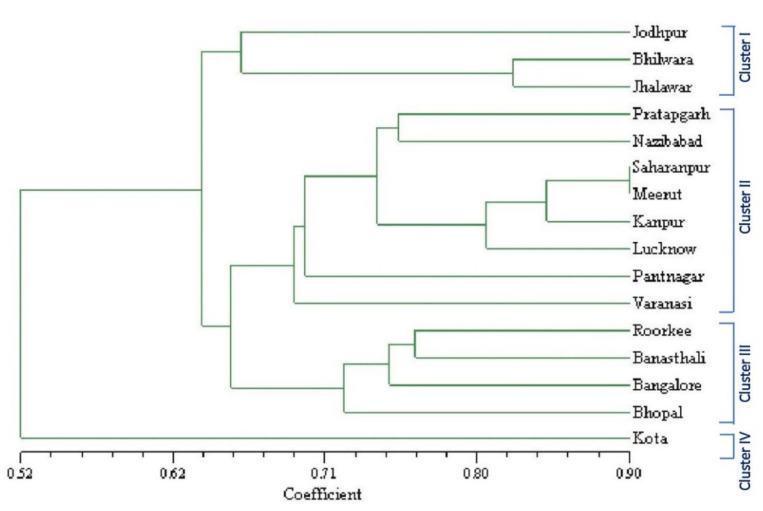
UPGMA dendrogram of 16 accessions of *S. cumini* based on 98 ISSR markers. Cluster I: Jodhpur, Bhilwara, and Jhalawar; Cluster II: Pratapgarh, Nazibabad, Saharanpur, Meerut, Kanpur, Lucknow, Pantnagar, and Varanasi; Cluster III: Roorkee, Banasthali, Bangalore, and Bhopal; Cluster IV: Kota.

The UPGMA method was used to build a dendrogram based on the resemblance matrix expressing Jaccard’s coefficient to assess the relationship between the genotypes. The dendrogram (Figure 2) shows that 16 genotypes from various places created four clusters. However, not even one of the discrete primers could group these plants into agroclimatic sub-zone or region-specific clusters. Cluster one was represented by Jodhpur (E-1.2), Bhilwara (D-4.2), and Jhalawar (D-4.2) accessions, which belonged to arid and semi-arid zones. Cluster two had samples from Pratapgarh (D-4.2), Nazibabad (CD-6.1), Saharanpur (CD-5.4), Meerut (CD-4.3), Kanpur (CD5.4), Lucknow (CD-5.4), Pantnagar (CM-6.2) and Varanasi (CD-4.1) and these showed mixing as samples belonged to semi-arid, moist sub-humid and dry-sub humid zones. Cluster three was represented by Roorkee (CD-6.1), Banasthali (D-3.3), Bangalore (D-4.4), and Bhopal (D-4.2). Except for Roorkee, all samples in this group fitted to the semi-arid agro-ecological central zone and three agro-climatic subzones. One accession, Kota (D-4.2), stood in for cluster 4 and was unique from all other accessions.

**Figure 3:**
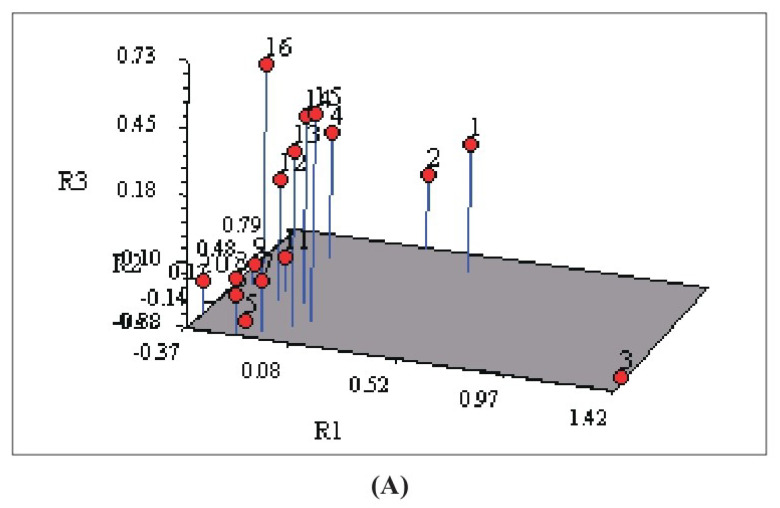

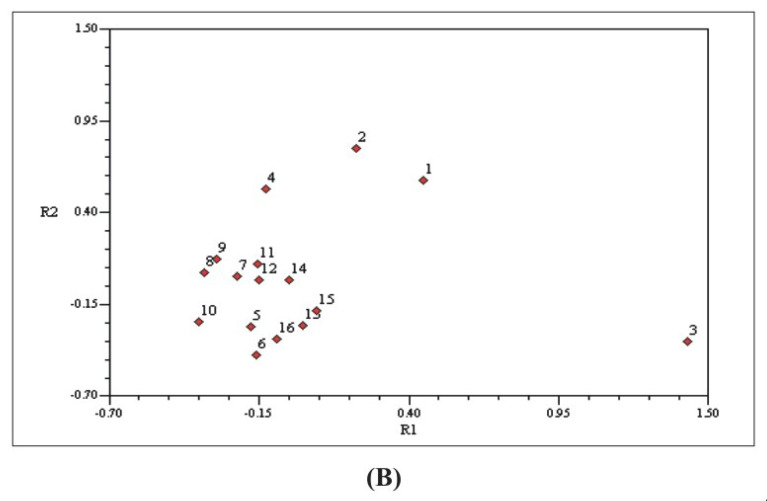
Three–dimensional (**A**) and two-dimensional (**B**) plots of principal component analysis based on ISSR in 16 *S. cumini* populations. The numbers are plotted to represent individual genotypes.

Despite the substantial diversity among the samples, UPGMA analysis did not reveal grouping built on areas or agro-climatic sub-zones. Apart from cluster analysis, the principal component analysis (PCA) was performed on group accessions (Figure 3). Clustering was useful in detecting relationships among ecotypes, while PCA allowed a view of the relationships between groups. The genetic difference did not exhibit any geographical pattern, and there was no discernible relationship between genetic and geographic distance, according to the PCA and UPGMA dendrogram.

#### 3.1.4. Shannon Diversity Index Based upon ISSR Markers

Based upon ISSR variation, Table 5 presents the Shannon phenotypic diversity for *S. cumini* populations. The H_ο_ within the semi-arid region ranged from 1.17 to 4.09, with a mean value of 2.65. Similarly, the lower and higher values of H_ο_ for dry sub-humid and moist sub-humid varied from 0.69 to 2.16 and 1.18 to 2.56, respectively. The means of H_ο_ for these two regions are 1.82 and 2.04, correspondingly. The H_ο_ might not be analyzed for the arid zone, as only one sample was from this district. The average diversity at a population level (Hcol) varied from a lower value of 1.03 to a higher value of 1.86 with a mean value of 1.54, whereas the observed genetic diversity at the species level (Hsp) ranged from 2.48 to 3.98 with a mean value of 3.00. A comparable value of diversity at the species level (4.1 to 7.9 with a mean value of 6.07) is reported in Mulberry [24].

The coefficient of genetic differentiation (Gst) varied from a lower value of 0.38 to a higher value of 0.58 with an average of 0.46, which specified a high degree of genetic differentiation among populations. It presented that 46% of genetic variation exists among populations and 54% within the inhabitants. For woody plants, wind and cross-pollination accounted for 10% of genetic diversity among communities [25]. The estimates were greater than the average for several reasons, including the longer geographic distance between the four groups and natural environmental selection.

Nm, or the degree of gene flow, was only calculated to be 0.30. The result is comparable with earlier studies, as the reported value of gene flow was 0.44 in *Changium smyrmiodes* [26] and 0.24 in *Eurya nitida* [27]. In contrast, relatively higher values were reported as 0.6 in *Phaseolus* [28] and 0.91 in *Mikania* [29]. The little gene flow between populations may cause significant genetic divergence in this species. A lower level of gene flow is further supported by the high partitioning of heterozygosity in groups and could be exploited at intra and inter-group levels. Geographical environments, ecological considerations, and the plant’s mating system likely shaped the increased genetic diversity within groups.

The estimates of Nm were 1.0 for *S. cumini* populations, indicating sufficient gene flow across populations to offset the effects of random drift. Genetic drift affects the genetic structure and heightens population divergence when populations are small and separate [30]. Consequently, reports of genetic divergence between populations have been made [31,32,33].

### 3.2. Phytochemical Analysis

#### 3.2.1. Total Phenol

**Figure 4:**
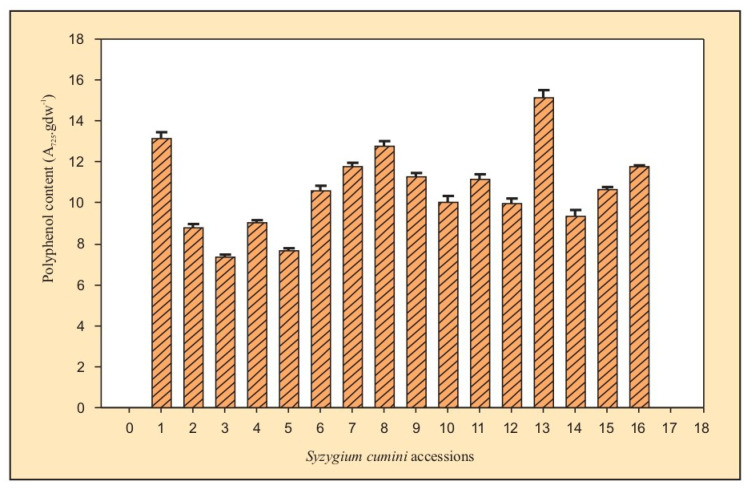
Total phenolic content in 16 *S. cumini* accessions.

Each plant’s leaf methanolic extract’s absorbance was measured at 725 nm, and the number of entire phenolics was calculated in terms of OD units. gdw^−1^. The Folin-Ciocalteu colorimetric assay in this study detects all hydroxylated phenolic compounds. The total phenolics in diverse samples of *S. cumini* are represented in Table 6; Figure 4. The highest level of total polyphenols was recorded in the Varanasi sample (15.11 OD units.gdw^−1^), followed by Jodhpur (13.16 OD units.gdw^−1^) sample. It is understandable because these samples belong to dry sub-humid and arid zones, with water stress conditions and stress plants producing higher polyphenol content [34].

The total phenolics in the samples that belonged to semi-arid regions were 8.77, 7.38, 9.04, 7.65, 10.57, 11.74, 12.75, 11.25 OD units.gdw^−1^ in Bhilwara, Kota, Jhalawar, Pratapgarh, Bhopal, Meerut, Banasthali, and Bangalore respectively. The total phenolics in the samples from the dry sub-humid region were 10.01, 11.12, 9.96, and 15.11 OD units.gdw^−1^ in Saharanpur, Kanpur, Lucknow, and Varanasi, respectively. In the moist sub-humid region samples, the phenolic content was higher, i.e., 9.36, 10.62, and 11.77 OD units.gdw^−1^ in Pantnagar, Nazibabad, and Roorkee, respectively. The polyphenol levels were particularly low in Pratapgarh, where total phenolics were only 7.65 OD units.gdw^−1^. Thus, substantial differences in total phenolics between diverse accessions of *S. cumini* were observed, although the variations within various regions were relatively low. The dry sub-humid region showed a relatively broader range of 9.96–15.11 OD units.gdw^−1^ in total phenolics. The distribution of total phenolics among 16 *S. cumini* accessions revealed that the arid zone had the highest concentration of phenolics, followed by moist sub-humid, semi-arid, and dry sub-humid regions. The varying amount of total phenolics from the leaves of *S. cumini* may be a function of the vegetational stage, the geographic position of plants, and climatic circumstances [35,36].

#### 3.2.2. Qualitative Analysis of Total Phenols Using TLC

Out of various tested solvent systems, 1, 5, 8, and 9 separated the standards of five phenolic acids, viz. ferulic acid, caffeic acid, sinapic acid, o-coumaric acid, and p-coumaric acid. The typical phenolic acid combination was divided into solvent system-1, which included benzene, acetic acid, and water, out of all the examined solvent systems (37:45:18). Table 7 lists the Rf values for several phenolic acid standards in various solvent arrangements.

Based on the better resolution, the samples were permissible to run in solvent systems 1 and 8. After treatment with FeCl_3_, a blackish-green and yellow spot was distinctly observed in *S. cumini*. P-coumaric acid and caffeic acid were separated in solvent 8. Consequently, in the qualitative analysis of total phenolics using preparative TLC, only caffeic acid and p-coumaric acid were tentatively recognized in around accessions of *S. cumini* samples.

#### 3.2.3. Qualitative Analysis of Phenolic Acids Using HPLC

The HPLC analysis showed variations in the qualitative and quantitative distribution of tentatively identified p-coumaric acid, caffeic acid, ferulic acid, and other unidentified compounds in different accession of *S. cumini*. The HPLC chromatograms of the five phenolic acids (ferulic acid, caffeic acid, p-coumaric acid, o-coumaric acid, and sinapic acid) and standard mixtures were monitored at 320 nm (Supplementary Figure S3). The retention time (RT) and areas of peaks for the standard phenolic acids used are given in Table 8. The RT of caffeic acid, ferulic acid, sinapic acid, o-coumaric acid, and p-coumaric acid were 6.88, 9.4, 9.07, 9.09, and 12.32, respectively.

Comparing RT with available standards, the compounds as peak numbers 3, 4, and 8 (RT 9.4, 9.1, and 6.8) were tentatively identified as ferulic acid, p-coumaric, and caffeic acid, respectively. For further confirmation, the sample from Varanasi and Jhalawar was co-chromatographed with caffeic acid and ferulic acid, respectively. The co-chromatography with authentic phenolic acid showed that the chromatograms’ peaks were separated with a simultaneous increase in the integration area value. Moreover, a significant amount of other phenolic compounds was also present, but these could not be further identified.

The concentration of each compound (identified/unidentified) was calculated based on peak area. Detailed quantification of diverse phenolic compounds (mg.gdwt^−1^) is concise in Table 9. The caffeic acid, which varied in its amount from 0.56 mg.gdwt^−1^ in Bhilwara to 7.14 mg.gdwt^−1^ in Lucknow, was present in all samples. The p-coumaric acid varied from 0.02 mg.gdwt^−1^ in Bhilwara to 0.26 mg.gdwt^−1^ in Jodhpur and was also present in all samples of *S. cumini*. On the contrary, tentatively identified ferulic acid was present only in some samples and ranged from a lower amount of 0.04 mg.gdwt^−1^ in Jodhpur to a higher amount of 2.31 mg.gdwt^−1^ in Roorkee. The ferulic acid yield was exceptionally high in Roorkee.

Based on their quantity, peaks 3 and 4 (RT 4.4 and 5.5) gave major and clear peaks of polyphenols with a relatively much higher percentage of area. The amount of unidentified compound as peak number 3 varied from 0.54 mg.gdwt^−1^ in Bhilwara (semi-arid) to 6.63 mg.gdwt^−1^ in Nazibabad (moist sub-humid). The peak 4 of the unknown compound originated at RT 5.5 and was enormous and ranged from 0.58 mg.gdwt^−1^ in Bhilwara (semi-arid) to 6.26 mg.gdwt^−1^ in Nazibabad (moist sub-humid). These two compounds were present in all accessions of *S. cumini*. Moreover, the other unidentified compounds as peak numbers 1, 2, 5, 6, 7, 9, 10, 11, 12 (RT 3.6, 3.9, 6.1, 6.3, 6.5, 7.3, 8.1, 8.4, and 8.6) were also observed in relatively lower but significant amount and were not presented in all samples.

Phenolic acids commonly found in wild medicinal plants are mainly caffeic acid, ferulic acid, p-coumaric, and sinapic acid. A comparable amount of caffeic acid and p-coumaric acid, 0.22 mg.g^−1^ and 0.77 mg.g^−1,^ respectively, was reported in *Eucalyptus uropendis* [37]. A study showed the concentration of caffeic acid (2.4 to 11.4 mg/L) and p-coumaric acid (0.0 and 3.0 mg/L) in different cultivars of pear juice [38]. On the contrary, lower amounts of caffeic acid (0–0.4-mg.g^−1^) were reported in aromatic herbs [39]. Similarly, high values of p-coumaric acid 0.6 (mg/L) and caffeic acid 5.1 (mg/L) were also reported in apple juices [40]. Table 10 shows the concentration of caffeic acid, ferulic acid, and p-coumaric acid in different accessions of *S. cumini*.

A helpful technique for categorization of the ”polyphenolic fingerprint” could be used [41]. It is characteristic of most plants because each species has a unique phenolic composition frequently used in chemotaxonomy [42,43]. There is no significant distribution pattern observed. However, specific chemotypes within the studied population of *S. cumini* from different geographic areas may be used in the manufacturing of phytomedicine for predicting phytochemical content in cultivar development. Moreover, it would also be helpful for plant breeding, quality control, and intellectual property rights (IPR).

### 3.3. Correlation between Phytochemical and Molecular Profiling

**Figure 5:**
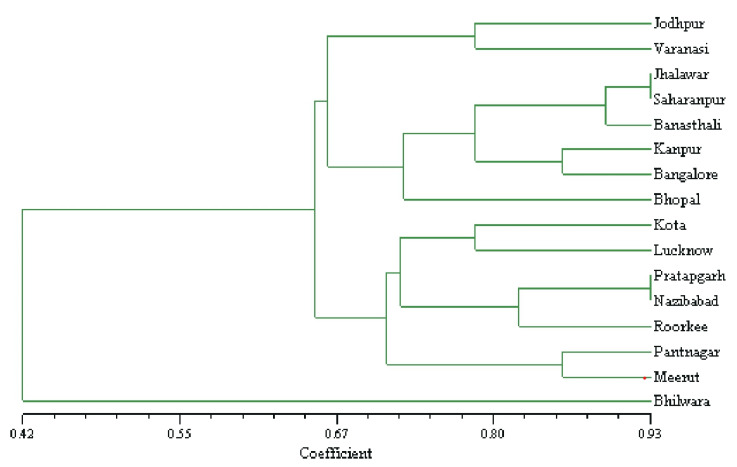
UPGMA dendrogram of the genetic relationships among 16 *S. cumini* genotypes based on HPLC.

The focus of this study was to check the correlation of molecular and phytochemical characters at the intraspecific level. Phytochemical profiling was employed as the base marker for chemotypic clustering. The dendrogram based on chemical profiling is shown in Figure 5. The dendrogram based on chromatogram grouped the samples into two major clusters with two samples as independent branches. As can be seen from the dendrogram, the grouping of samples did not follow any pattern related to their agro-ecological region or geographical distance. The dendrogram based on chromatogram was compared with that generated using the molecular technique ISSR and showed little correlation. Samples from Pratapgarh and Banasthali, Chittorgarh, and Roorkee showed similarity in chemotyping and ISSR. Despite some similarities, a distinct correlation between the grouping of samples based on chemoprofiling and molecular profiling could not be established.

A study conducted in 2005 also could not correlate the genetic dendrogram with that based on essential oil content in *Cymbopogon* sp. [44]. This is understandable as chemoprofiling is influenced by the related climatic condition, geographic location, and vegetational stage of plants and leaves [44], whereas molecular profiling completely depicts the genetic structure of the plant. Conversely, a close relation was also reported in chemical and genetic variation of the *Vitex rotundifolia* population from different locations in China [45]. In 2006, another study reported a strong correlation between secondary metabolite content and the genetic characterization of six *Hypericum* species from Serbia [46].

The presented data imply that the morphotypes are of multiple origins or due to different ecological growing conditions rather than genetically determined and that phytochemical races are induced by a limited number of genetic differences, which might have occurred independently in different *S. cumini* populations. Analysis of genetic profiles, the type and content of polyphenols produced in each of the accessions of *S. cumini* could constitute valuable tools for analyzing this specie’s biodiversity. Since these analyses successfully discriminate between the accessions of this species, they constitute interesting tools to select those accessions with the potential to be used for specific crop improvement programs. We explored the use of these analyses performed on different accessions of the species collected from geographically distinct areas of India. Our findings can be used for the commercial production and germplasm management of this medicinal plant.

## 4. Conclusions

It has been well-documented that geographical conditions may affect the phytochemical profile of the plant. In addition to genetic research, phytochemical differences are investigated as a method for evaluating biodiversity and authenticating germplasm. This investigation revealed a large amount of variation across *S. cumini* accessions. The most effective accessions for improvement projects were identified using the ISSR marker system and the study of commercially significant phenolic acids. These accessions might be employed as acceptable feedstock for various commercial uses.

## Figures and Tables

**Table 1:** Details of *S. cumini* samples gathered from diverse agro-ecological zones of India.

S. No.	Major Zones	Sub Zones	Place	Sample Code	State	Latitude	Longitude
1.	Arid	E-1.2	Jodhpur	JD1	Rajasthan	N 26°23′	E 73°8′
2.	Semi-arid	D-4.2	Bhilwara	JD2	Rajasthan	N 22°25′	E 74°38′
3.		D-4.2	Kota	JD3	Rajasthan	N 24°14′	E 75°49′
4.		D-4.2	Jhalawar	JD4	Rajasthan	N 24°40′	E 76°10′
5.		D-4.2	Pratapgarh	JD5	Rajasthan	N 24°20′	E 74°40′
6.		D-4.2	Bhopal	JD14	MP	N 23°20′	E 77°30′
7.		D-4.3	Meerut	JD9	UP	N 29°10′	E 77°42′
8.		D-3.3	Banasthali	JD15	Rajasthan	N 26°60′	E 75°54′
9.		D-4.4	Bangalore	JD16	Karnataka	N 12°59′	E 77°40′
10.	Dry sub-humid	CD-5.4	Saharanpur	JD7	UP	N 29°58′	E 77°33′
11.		CD-5.4	Kanpur	JD8	UP	N 26°28′	E 80°20′
12.		CD-5.4	Lucknow	JD11	UP	N 26°50′	E 81°00′
13.		CD-4.1	Varanasi	JD12	UP	N 25°22′	E 83°00′
14.	Moist sub-humid	CM-6.2	Pantnagar	JD6	Uttrakhand	N 29°31′	E 79°30′
15.		CD-6.1	Nazibabad	JD10	Uttrakhand	N 29°36′	E 78°18′
16.		CD-6.1	Roorkee	JD14	Uttrakhand	N 29°52′	E 77°59′

**Table 2:** ISSR primers with their sequence and calculated Tm.

S. No.	Primer Code	Oligomer Length (bp)	Tm	Primer Sequence (5’-3′)
1.	15BV17C1	17	52	(AG)8 C
2.	16BV17A2	17	50	(GA)8A
3.	17BV18T3	18	52	(GA)8YT
4.	18BV18C4	18	54	(GA)8YC
5.	19BV19C5	19	55	(AC)8YAC
6.	20BV17A6	17	56	(AGC)5CA
7.	21BV17G7	17	53	(AGC)5GC
8.	22BV17T8	17	56	(AGC)5GT
9.	23BV17C9	17	58	(AGC)5GC
10.	24BV16T10	16	46	(CA)7AT
11.	25BV16C11	16	48	(CA)7AC
12.	26BV16T12	16	46	(CA)7GT
13.	27BV16C13	16	50	(CA)7GC
14.	28BV16A14	16	48	(CA)7GA
15.	29BV16A15	16	46	(CA)7AA
16.	30BV16A16	16	46	(GT)7TA
17.	31BV16G17	16	48	(GT)7TG
18.	32BV16A18	16	48	(GT)7CA
19.	33BV16T19	16	48	(GT)7CT
20.	34BV16T20	16	46	(GT)7AT
21.	35BV16C21	16	48	(GT)7AC
22.	36BV17A22	18	52	GCTG(AG)6 A
23.	37BV17C23	17	52	(GA)8C
24.	38BV17C24	17	52	(AC)8C
25.	39BV19C25	19	58	(AG)8 GCTC
26.	40BV17T26	17	50	(GA)7CTT

**Table 3:** Quantification of genomic DNA of *S. cumini* leaf samples collected from different places using spectrophotometer and gel documentation system.

S. No.	Places	Spectrophotometric Based			Gel Doc Based		
		λ260\λ280	Conc. μg.μL^−1^	Yield μg.gdw^−1^	MW (Kb)	Conc. μg.μL^−1^	Yield μg.gdw^−1^
1.	Jodhpur	1.81	3.0	300	20.4	1.07	214
2.	Bhilwara	1.92	2.5	250	19.8	1.03	206
3.	Kota	1.97	3.0	300	20.4	0.65	130
4.	Jhalawar	2.00	3.3	330	20.4	0.94	188
5.	Pratapgarh	1.82	2.6	260	19.5	0.96	192
6.	Pantnagar	2.02	3.5	350	19.5	0.81	162
7.	Saharanpur	1.86	3.2	320	19.5	0.85	170
8.	Kanpur	1.91	3.8	380	19.5	0.97	194
9.	Meerut	2.16	3.2	320	19.6	0.95	190
10.	Nazibabad	1.74	2.6	260	20.4	0.82	164
11.	Lucknow	1.83	3.8	380	20.4	0.75	150
12.	Varanasi	2.00	2.1	210	20.4	1.16	232
13.	Roorkee	1.80	4.1	410	20.4	1.13	226
14.	Bhopal	2.06	1.9	190	19.5	0.92	184
15.	Banasthali	1.93	2.3	230	20.4	0.77	154
16.	Bangalore	2.04	3.2	320	19.5	0.95	190

**Table 4:** ISSR primers employed in the genetic diversity of *S. cumini* germplasm with their corresponding percentage polymorphism, gene diversity, and resolving power.

S. No.	Primer Code	Sequence (5′-3′)	Total no. of the Amplified Band (NAB)	Total no. of Polymorphic Fragments (NPB)	% Polymorphic Band (PPB)	Resolving Power	Gene Diversity (PIC)
1.	15BV17 C1	(AG)_8_ C	15	15	100.00	14.00	0.61
2.	17BV18 T3	(GA)_8_YT	17	16	94.11	11.5	0.65
3.	18BV18C4	(GA)_8_YC	11	10	90.90	8.00	0.74
4.	20BV17 A6	(AGC)_5_CA	12	12	100.00	6.25	0.77
5.	22BV17 T8	(AGC)_5_GT	7	7	100.00	6.50	0.75
6.	26BV16 T12	(CA)_7_GT	11	11	100.00	11.00	0.68
7.	27BV16 C13	(CA)_7_GC	11	10	90.90	9.12	0.73
8.	40BV17 T26	(GA)_7_CTT	14	14	100.00	10.50	0.84
	Average		12.25	11.87	96.25	10.08	0.74

**Table 5:** Genetic diversity in *S. cumini* germplasm and Shannon diversity index based on ISSR markers.

Primer Code	Sequence (5′-3’)	H Arid	H Semi-Arid	H Dry Sub-Humid	H Moist Sub-Humid	Hcol	Hcol/Hsp	Gst = (Hsp-Hcol)/Hsp	Hsp
15BV17 C1	(AG)_8_ C	-	3.22	1.81	1.46	1.62	0.47	0.52	3.44
17BV18 T3	(GA)_8_YT	-	3.21	1.68	1.18	1.69	0.54	0.45	3.12
18BV18C4	(GA)_8_YC	-	1.17	0.69	2.28	1.03	0.41	0.58	2.48
20BV17A6	(AGC)_5_CA	-	2.27	1.73	2.56	1.22	0.46	0.53	2.65
22BV17T8	(AGC)_5_GT	-	2.38	2.16	1.18	1.43	0.62	0.37	2.30
26BV16T12	(CA)_7_GT	-	2.42	2.03	2.92	1.84	0.61	0.38	3.00
27BV16C13	(CA)_7_GC	-	2.45	1.94	2.28	1.67	0.55	0.44	3.02
40BV17T26	(GA)_7_CTT	-	4.09	1.38	2.46	1.86	0.61	0.38	3.98
Average		-	**2.65**	**1.82**	**2.04**	**1.54**	**0.53**	**0.46**	**3.00**
Nm									**0.30**

H = Wthin-collection genetic diversity per primer, Hο = Mean within collection genetic diversity, Hcol = Mean within-collection genetic diversity for all collections, Hsp = Genetic diversity of the species, Hcol/Hsp = percentage of diversity within collections, Gst = [Hsp-Hcol]/Hsp = Diversity between collections, Nm = Gene flow.

**Table 6:** Total polyphenol content * in various *S. cumini* populations.

S. No.	Major Zones	Sub-Zones	Place	Sample Code	Yield (A_725_.gdw^−1^) *
1.	Arid	E-1.2	Jodhpur	JS1	13.16 ± 0.31
2.	Semi-arid	D-4.2	Bhilwara	JS2	8.77 ± 0.22
3.		D-4.2	Kota	JS3	7.38 ± 0.11
4.		D-4.2	Jhalawar	JS4	9.04 ± 0.12
5.		D-4.2	Pratapgarh	JS5	7.65 ± 0.16
6.		D-4.2	Bhopal	JS13	10.57 ± 0.29
7.		D-4.3	Meerut	JS9	11.74± 0.23
8.		D-3.3	Banasthali	JS15	12.75 ± 0.27
9.		D-4.4	Bangalore	JS16	11.25 ± 0.21
10.	Dry sub-humid	CD-5.4	Saharanpur	JS7	10.01 ± 0.34
11.		CD-5.4	Kanpur	JS8	11.12 ± 0.29
12.		CD-5.4	Lucknow	JS11	9.96 ± 0.23
13.		CD-4.1	Varanasi	JS12	15.11 ± 0.37
14.	Moist sub-humid	CM-6.2	Pantnagar	JS6	9.36 ± 0.28
15.		CD-6.1	Nazibabad	JS10	10.62 ± 0.15
16.		CD-6.1	Roorkee	JS14	11.77 ± 0.07

* The values represent the mean ± SD with *n* = 3.

**Table 7:** Rf value of standards in different solvents *.

S. No.	Phenolic Acid	Solvent 1	Solvent 5	Solvent 8	Solvent 9
1.	Caffeic acid	0.36	0.84	0.87	0.91
2.	p-Coumaric acid	0.54	0.88	0.90	0.88
3.	o-Coumaric acid	0.50	0.84	0.85	0.86
4.	Sinapic acid	0.59	0.88	0.87	0.86
5.	Ferulic acid	0.68	0.90	0.80	0.88

* Solvent system 1 (Benzene: acetic acid: water = 37:45:8); 5 (Ethyl acetate: toluene: acetic acid = 50:40:20); 8 (Ethyl acetate: acetic acid = 80:20); 9 (Ethyl acetate: methanol = 80:20).

**Table 8:** Standard phenolic acids retention time (RT) and peak area.

S. No.	Standard Phenolic Acids	Retention Time (RT)	Area
1.	Caffeic acid	6.88	7,642,471
2.	Ferulic acid	9.40	8,017,806
3.	Sinapic acid	9.07	8,801,563
4.	p-Coumaric acid	9.09	11,156,442
5.	o-Coumaric acid	12.32	10,651,248
Total Area			46,269,530

**Table 9:** Quantification of total phenolics in *S. cumini* (mg.gdwt^–1^) accessions.

			Arid	Semi-Arid								Dry Sub-Humid				Moist Sub-Humid		
Peak No.	Retention Time	Probable Identity	Jodhpur	Bhilwara	Kota	Jhalawar	Pratapgarh	Bhopal	Meerut	Banasthali	Bangalore	Saharanpur	Kanpur	Lucknow	Varanasi	Pantnagar	Nazibabad	Roorkee
1.	3.6	-	0.01	0.07	0.08	0.08	-	0.31	0.01	-	-	0.02	0.03	0.21	0.01	0.05	-	-
2.	3.9	-	0.10	0.04	-	0.08	0.65	-	0.08	40.47	142.51	0.09	0.11	-	70.11	0.04	0.25	241.06
3.	4.4	-	3.29	0.54	1.47	1.70	3.56	1.55	1.41	2.44	5.48	1.70	3.18	1.75	2.20	1.70	6.63	6.36
4.	5.5	-	1.55	0.58	1.19	2.45	5.04	1.16	1.25	1.70	2.04	1.88	2.87	4.22	1.87	2.54	6.26	3.90
5.	6.1	-	0.26	-	0.02	0.72	0.33	-	0.41	0.41	0.20	0.12	-	0.04	1.68	0.07	2.07	-
6.	6.3	-	0.06	-	0.08	-	0.04	-	-	-	-	-	-	0.04	0.13	0.25	-	0.20
7.	6.5	-	0.04	0.06	-	0.18	-	0.08	-	0.11	0.04	0.09	1.93	-	0.13	-	-	-
8.	6.8	Caffeic Acid	1.67	0.56	3.63	5.00	1.42	1.60	5.14	3.58	1.69	2.04	3.94	7.14	5.65	2.47	1.87	1.60
9.	7.3	-	0.13	0.06	0.08	0.24	0.45	0.30	0.20	0.06	0.28	0.30	0.85	0.12	0.22	0.35	0.67	1.26
10.	8.1	-	0.05	0.06	1.67	0.07	0.06	0.30	0.08	0.06	-	0.03	-	0.02	0.01	-	0.12	0.08
11.	8.4	-	0.01	0.05	-	0.10	0.41	0.01	-	0.01	-	0.06	0.01	-	-	0.01	-	-
12.	8.6	-	0.01	0.02	-	-	-	0.17	-	-	-	-	0.01	-	0.04	0.01	-	-
13.	9.1	p-Coumaric	0.26	0.02	0.20	0.02	0.04	0.05	0.06	0.03	0.06	0.03	0.02	0.12	0.03	0.10	0.03	0.07
14.	9.4	Ferulic Acid	0.04	0.12	-	0.08.	-	0.06	-	-	-	-	-	0.12	0.14	-	-	2.31

**Table 10:** The concentration (mg/100 g.dw^−1^) of phenolic acids (caffeic acid, ferulic acid, and p-coumaric acid) in *S. cumini* accessions.

S. No.	Sample	Caffeic Acid	Ferulic Acid	p-Coumaric Acid
1.	Jodhpur	167.71	4.65	26.79
2.	Bhilwara	56.66	11.96	2.03
3.	Kota	363.04	nd	20.27
4.	Jhalawar	500.96	0.85	2.71
5.	Pratapgarh	142.15	nd	4.42
6.	Pantnagar	247.26	nd	9.08
7.	Saharanpur	204.17	nd	3.59
8.	Kanpur	39.47	nd	2.91
9.	Meerut	514.06	nd	6.85
10.	Nazibabad	187.67	nd	3.71
11.	Lucknow	714.04	12.62	12.27
12.	Varanasi	565.76	14.80	3.29
13.	Roorkee	160.72	231.40	7.19
14.	Bhopal	160.27	0.69	5.57
15.	Banasthali	358.05	nd	3.66
16.	Banglore	169.07	nd	6.08

## Data Availability

Not applicable.

## Supplementary Materials

The following supporting information can be downloaded at: https://www.mdpi.com/article/10.3390/plants12040931/s1, Figure S1: ISSR pattern of *S. cumini* samples with primer 1, 2, 3, and 4. Lane 1 = Jodhpur, 2 = Bhilwara, 3 = Kota, 4 = Jhalawar, 5 = Pratapgarh, 6 = Pantnagar, 7 = Saharanpur, 8 = Kanpur, 19 = Meerut, 10 = Nazibabad, 11 = Lucknow, 12 = Varanasi, 13 = Roorkee, 14 = Bhopal, 15 = Banasthali, 16 = Bangalore, M = Molecular weight marker (Low range DNA ruler); Figure S2: ISSR pattern of *S. cumini* samples with primer 5, 6, 7 and 8. Lane 1 = Jodhpur, 2 = Bhilwara, 3 = Kota, 4 = Jhalawar, 5 = Pratapgarh, 6 = Pantnagar, 7 = Saharanpur, 8 = Kanpur, 19 = Meerut, 10 = Nazibabad, 11 = Lucknow, 12 = Varanasi, 13 = Roorkee, 14 = Bhopal, 15 = Banasthali, 16 = Bangalore, M = Molecular weight marker (Low range DNA ruler); Figure S3: HPLC chromatograms of the five phenolic acids. A = Caffeic acid, B = Ferulic acid, C = Sinapic acid, D = p-Coumaric acid, E = o-Coumaric acid, and F = Co-chromatography of all five standards, peak 1, 2, 3, and 4 represent caffeic acid, ferulic acid, p-coumaric/sinapic acid, and o-coumaric acid respectively.

## References

[1] Brummitt N.A., Bachman S.P., Griffiths-Lee J., Lutz M., Moat J.F., Farjon A., Donaldson J.S., Hilton-Taylor C., Meagher T.R., Albuquerque S. (2015). Green Plants in the Red: A Baseline Global Assessment for the IUCN Sampled Red List Index for Plants. PLoS ONE.

[2] Pironon S., Borrell J.S., Ondo I., Douglas R., Phillips C., Khoury C.K., Kantar M.B., Fumia N., Soto Gomez M., Viruel J. (2020). Toward unifying global hotspots of wild and domesticated biodiversity. Plants.

[3] He F., Kang D., Ren Y., Qu L.J., Zhen Y., Gu H. (2007). Genetic diversity of the natural populations of *Arabidopsis thaliana* in China. Heredity.

[4] Madani B., Mirshekari A., Yahia E.M., Golding J.B., Hajivand S., Dastjerdy A.M. (2021). Jamun (*Syzygium cumini* L. Skeels): A Promising Fruit for the Future. Hortic. Rev..

[5] Singh J.S., Chaturvedi R.K. (2017). Diversity of Ecosystem Types in India: A Review. Proc. Indian Natl. Sci. Acad..

[6] Sardar N.R., Akbari S.H., Bhatt H.G., Tagalpallewar G.P. (2022). Chemical and mineral composition of jamun fruit pulp (*Syzygium cumini* L.). Pharma Innov..

[7] De Carvalho J.A., Beale M.A., Hagen F., Fisher M.C., Kano R., Bonifaz A., Toriello C., Negroni R., Rego R.D.M., Gremião I.D.F. (2021). Trends in the molecular epidemiology and population genetics of emerging *Sporothrix* species. Stud. Mycol..

[8] Zietkiewicz E., Rafalski A., Labuda D. (1994). Genome Fingerprinting by Simple Sequence Repeat (SSR)-Anchored Polymerase Chain Reaction Amplification. Genomics.

[9] Pianzzola M.J., Zarantonelli L., González G., Fraguas L.F., Vázquez A. (2004). Genetic, phytochemical and biochemical analyses as tools for biodiversity evaluation of wild accessions of *Solanum commersonii*. Biochem. Syst. Ecol..

[10] Jiang H., Xie Z., Koo H.J., McLaughlin S.P., Timmermann B.N., Gang D.R. (2006). Metabolic profiling and phylogenetic analysis of medicinal Zingiber species: Tools for authentication of ginger (*Zingiber officinale* Rosc.). Phytochemistry.

[11] Grass S., Zidorn C., Blattner F.R., Stuppner H. (2006). Comparative molecular and phytochemical investigation of *Leontodon autumnalis* (Asteraceae, Lactuceae) populations from Central Europe. Phytochemistry.

[12] Pandey A., Belwal T., Tamta S., Bhatt I.D., Rawal R.S. (2019). Phenolic compounds, antioxidant capacity and antimutagenic activity in different growth stages of in vitro raised plants of *Origanum vulgare* L. Mol. Biol. Rep..

[13] Khan S., Sharma V. (2010). Genetic differentiation and diversity analysis of medicinal tree *Syzygium cumini* (Myrtaceae) from eco-logically different regions of India. Physiol. Mol. Biol. Plants.

[14] Jana S., Shekhawat G.S. (2012). In vitro regeneration of Anethum graveolens, antioxidative enzymes during organogenesis and RAPD analysis for clonal fidelity. Biol. Plant.

[15] Hung C.-Y., Yen G.-C. (2002). Antioxidant Activity of Phenolic Compounds Isolated from *Mesona procumbens* Hemsl. J. Agric. Food Chem..

[16] Rohlf F.J. (1993). NTSYS-Pc Numerical Taxonomy and Multivariate Analysis System, Version 2.1.

[17] Jaccard P. (1908). Nouvelles recherches sur la distribution florale. Bull. Soc. Vaud. Sci. Nat..

[18] Prevost A., Wilkinson M.J. (1999). A new system of comparing PCR primers applied to ISSR fingerprinting of potato cultivars. Theor. Appl. Genet..

[19] Anderson J.A., Churchill G.A., Autrique J.E., Tanksley S.D., Sorrells M.E. (1993). Optimizing parental selection for genetic linkage maps. Genome.

[20] Powell W., Morgante M., Andre C. (1996). The comparision of RFLP, RAPD, AFLP and SSR (microsatellite) marker for germplasm analysis. Mol. Breed..

[21] Singh V.K., Avtar R., Mahavir N.K., Manjeet R.K., Rathore V. (2020). Assessment of genetic relationship among diverse Indian mustard (*Brassica juncea* L.) genotypes using XLSTAT. Electron. J. Plant Breed..

[22] Kulkarni M., Borse T., Chaphalkar S. (2007). Isolation and purification of genomic DNA from Black Plum (*Eugenia jambolana* Lam.) for analytical applications. Int. J. Biotechnol. Biochem..

[23] Morgante M., Oliveri A.M. (1993). PCR-amplified microsatellites as markers in plant genetics. Plant J..

[24] Amel S.-H., Khaled C., Messaoud M., Mohamed M., Mokhtar T. (2005). Comparative Analysis of Genetic Diversity in Two Tunisian Collections of Fig Cultivars Based on Random Amplified Polymorphic DNA and Inter Simple Sequence Repeats Fingerprints. Genet. Resour. Crop. Evol..

[25] Hamrick J.L., Godt M.J.W. (1989). Allozyme diversity in plant species. Plant Population Genetics, Breeding and Genetic Resources, Brown, A.H.D., Clegg, M.T., Kahler, A., Eds.

[26] Qiu Y.-X., Hong D.-Y., Fu C.-X., Cameron K.M. (2003). Genetic variation in the endangered and endemic species *Changium smyrnioides* (Apiaceae). Biochem. Syst. Ecol..

[27] Bahulikar R.A., Lagu M.D., Kulkarmi B.G., Pandit S.S., Suresh H.S.R., Ranjekar M.K.V., Gupta V.S. (2004). Genetic diversity among spatially isolated populations of *Euriya nitida* Korth (Theacea) based on inter-simple sequences repeats. Curr. Sci..

[28] Cruz E.P., Gepts P., GarciaMarín P.C., Villareal D.Z. (2005). Spatial Distribution of Genetic Diversity in Wild Populations of *Phaseolus vulgaris* L. from Guanajuato and Michoacán, Méexico. Genet. Resour. Crop. Evol..

[29] Li J.-M., Jin Z.-X. (2007). Genetic structure of endangered *Emmenopterys henryi* Oliv. based on ISSR polymorphism and implications for its conservation. Genetica.

[30] Ellstrand N.C., Elam D.R. (1993). Population genetic consequences of small population size. Implications for plant conservation. Annu. Rev. Ecol. Syst..

[31] Sun M.K., Wong C. (2001). Genetic structure of three orchid species with contrasting breeding systems using RAPD and allozyme markers. Am. J. Bot..

[32] Li Q., Xu Z., He T. (2002). Ex situ genetic conservation of endangered *Vatica guangxiensis* (Dipterocarpaceae) in China. Biol. Conserv..

[33] Vaishali, Khan S., Sharma V. (2008). RAPD based assessment of genetic diversity of *Butea monosperma* from different agro-ecological regions of India. Indian J. Biotechnol..

[34] Pour Nikfardjam M.S., Márk L., Avar P., Figler M., Ohmacht R. (2006). Polyphenols, anthocyanins, and trans-resveratrol in red wines from the Hungarian Villány region. Food Chem..

[35] Gambelli L., Santaroni G. (2004). Polyphenols content in some Italian red wines of different geographical origins. J. Food Compos. Anal..

[36] Maksimović Z., Malenčić D., Kovačević N. (2005). Polyphenol contents and antioxidant activity of Maydis stigma extracts. Bioresour. Technol..

[37] Chapuis-Lardy L., Contour-Ansel D., Bernhard-Reversat F. (2002). High-performance liquid chromatography of water-soluble phenolics in leaf litter of three Eucalyptus hybrids (Congo). Plant Sci..

[38] Tanrıöven D., Ekşi A. (2005). Phenolic compounds in pear juice from different cultivars. Food Chem..

[39] Wang H., Provan G.J., Helliwell K. (2004). Determination of rosmarinic acid and caffeic acid in aromatic herbs by HPLC. Food Chem..

[40] Schieber A., Keller P., Carle R. (2001). Determination of phenolic acids and flavanoids of apple and pear by high performance liquid chromatography. J. Liq. Chromatogr. A.

[41] Palade L.M., Popa M.E. (2018). Polyphenol Fingerprinting Approaches in Wine Traceability and Authenticity: Assessment and Implications of Red Wines. Beverages.

[42] McKeehen J.D., Busch R.H., Fulcher R.G. (1999). Evaluation of wheat (*Triticum aestivum* L.) phenolic acids during grain development and their contribution to Fusarium resistance. J. Agri. Food Chem..

[43] Antolovich M., Prenzler P., Robards K., Ryan D. (2000). Sample preparation in the determination of phenolic compounds in fruits. Analyst.

[44] Khanuja S.P., Shasany A.K., Pawar A., Lal R., Darokar M., Naqvi A., Rajkumar S., Sundaresan V., Lal N., Kumar S. (2005). Essential oil constituents and RAPD markers to establish species relationship in Cymbopogon Spreng. (Poaceae). Biochem. Syst. Ecol..

[45] Hu Y., Hou T.T., Xin H.L., Zhang Q.Y., Zheng H.C., Rahman K., Qin L.P. (2007). Estrogen like activity of volatile components from *Vitex royundifolia* L. Indian J. Med. Res..

[46] Smelcerovic A., Verma V., Spiteller M., Ahmad S.M., Puri S.C., Qazi G.N. (2006). Phytochemical analysis and genetic character-ization of six Hypericum species from Serbia. Phytochemistry.

