# Supplementary material for: Molecular Fingerprinting and Phytochemical Investigation of Syzygium cumini L. from Different Agro-Ecological Zones of India
Source: Plants (Basel). 2023 Feb 17;12(4):931. doi: 10.3390/plants12040931 (PMC9961202; doi:10.3390/plants12040931)
Supplement: Supplementary file 1 [file plants-12-00931-s001.zip › plants-2198654-supplementary.pdf]

plants

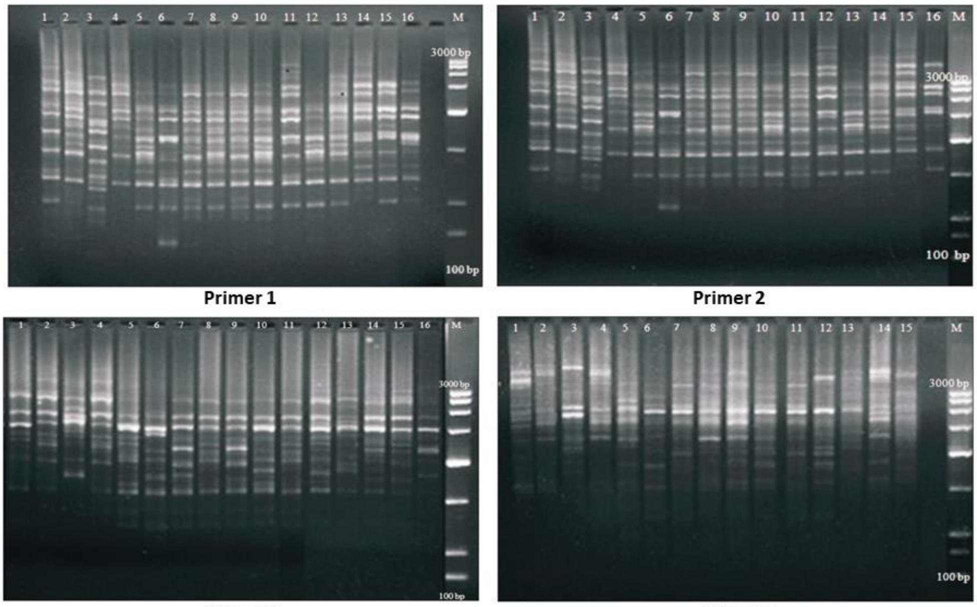

Figure S1. ISSR pattern of *S. cumini* samples with primer 1, 2, 3 and 4. Lane 1 = Jodhpur, 2 = Bhilwara, 3 = Kota, 4 = Jhalawar, 5 = Pratapgarh, 6 = Pantnagar, 7 = Saharanpur, 8 = Kanpur, 19 = Meerut, 10 = Nazibabad, 11 = Lucknow, 12 = Varanasi, 13 = Roorkee, 14 = Bhopal, 15 = Banasthali, 16 = Bangalore, M = Molecular weight marker (Low range DNA ruler).

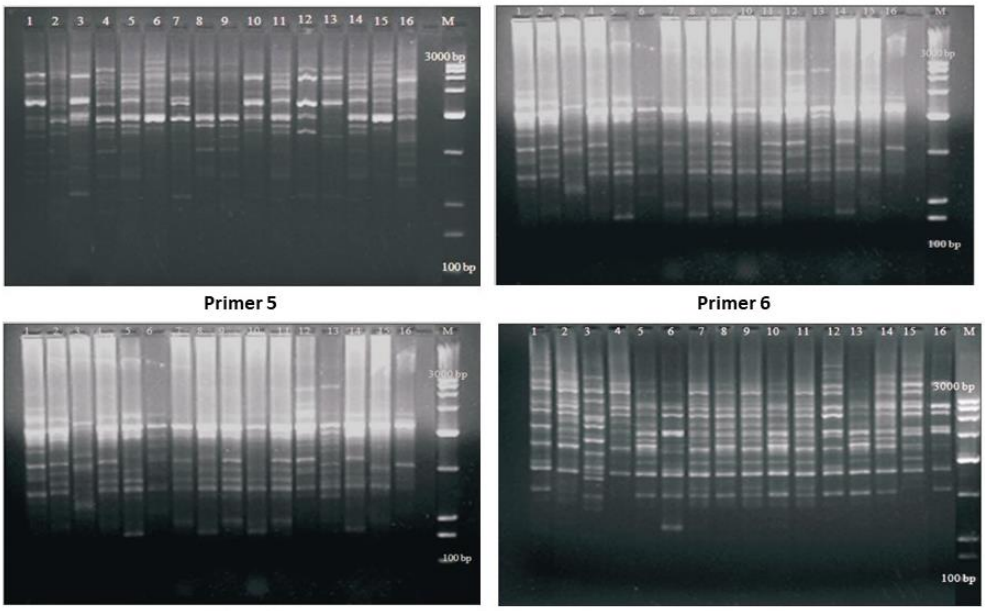

Figure S2. ISSR pattern of *S. cumini* samples with primer 5, 6, 7 and 8. Lane 1 = Jodhpur, 2 = Bhilwara, 3 = Kota, 4 = Jhalawar, 5 = Pratapgarh, 6 = Pantnagar, 7 = Saharanpur, 8 = Kanpur, 19 = Meerut, 10 = Nazibabad, 11 = Lucknow, 12 = Varanasi, 13 = Roorkee, 14 = Bhopal, 15 = Banasthali, 16 = Bangalore, M = Molecular weight marker (Low range DNA ruler).

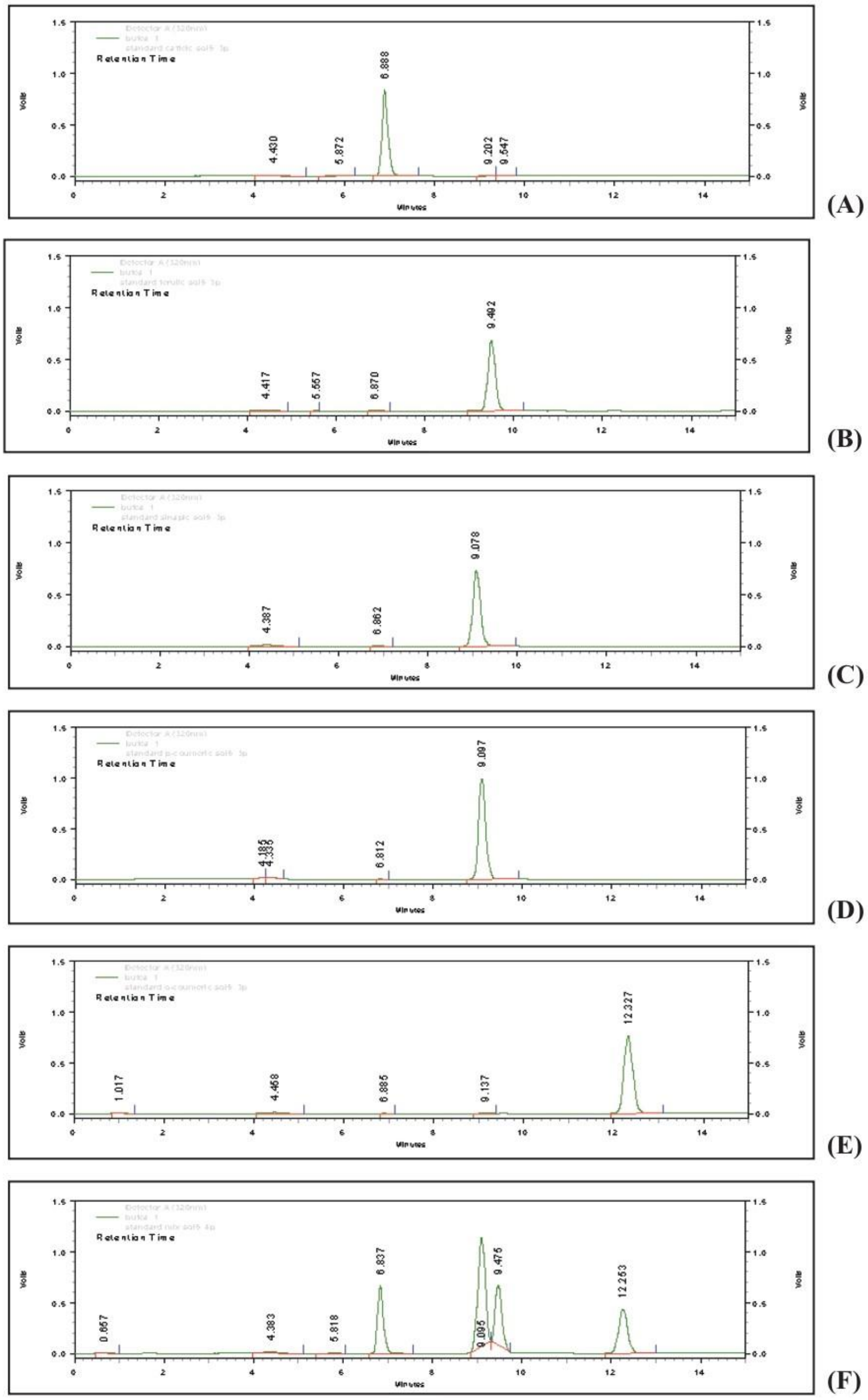

Figure S3. HPLC chromatograms of the five phenolic acids. A=Caffeic acid, B=Ferulic acid, C=Sinapic acid, D=p-Coumaric acid, E=o-Coumaric acid and F=Co-chromatography of all five standards, peak 1, 2, 3 and 4 represent caffeic acid, ferulic acid, p-coumaric/sinapic acid and o-coumaric acid respectively.
